# Machine Learning-Based Prediction of Three-Year Heart Failure and Mortality After Premature Ventricular Contraction Ablation

**DOI:** 10.3390/diagnostics15212693

**Published:** 2025-10-24

**Authors:** Chung-Yu Lin, Yu-Te Lai, Chien-Wei Chuang, Chih-Hsien Yu, Chiung-Yun Lo, Mingchih Chen, Ben-Chang Shia

**Affiliations:** 1 Graduate Institute of Business Administration, Fu Jen Catholic University, New Taipei City 242062, Taiwan; a02076@mail.fjuh.fju.edu.tw (C.-Y.L.); kanechuang2021@gmail.com (C.-W.C.);; 2 Department of Cardiology, Fu Jen Catholic University Hospital, New Taipei City 242062, Taiwan; 3 Department of Thoracic Medicine, St. Pual’s Hospital, Taoyuan City 330058, Taiwan; doc2098@mail.sph.org.tw; 4 Department of Cardiology, St Paul’s Hospital, Taoyuan City 330058, Taiwan; 5 Department of Teaching and Research, St. Paul’s Hospital, Taoyuan City 330058, Taiwan

**Keywords:** machine learning, artificial intelligence, PVC, catheter ablation surgery, risk factor, heart failure, good health and well-being

## Abstract

**Introduction:** Long-term heart failure and mortality after catheter ablation for premature ventricular contraction (PVC) remain underexplored. **Methods:** We retrospectively analyzed 4195 adults who underwent PVC ablation in a nationwide claims database. To address class imbalance, we used synthetic minority over-sampling technique (SMOTE) and random over-sampling examples (ROSE). Five supervised algorithms were compared: logistic regression, decision tree, random forest, XGBoost, and LightGBM. Discrimination was assessed by stratified five-fold cross-validation using the area under the receiver operating characteristic curve (ROC AUC). Because rare events can bias ROC, we also examined precision–recall (PR) curves. **Results:** For predicting three-year heart failure, LightGBM with ROSE achieved the highest ROC AUC at 0.822. For three-year mortality, logistic regression with ROSE and LightGBM with ROSE showed balanced performance with ROC AUCs of 0.886 and 0.882. Pairwise DeLong tests indicated that these leading models formed a high-performing cluster without significant differences in ROC AUC. Age, prior heart failure, malignancy, and end-stage renal disease were the most influential predictors by model explainability analysis. **Discussion:** Addressing class imbalance and benchmarking modern learners against a transparent logistic baseline yielded robust, clinically interpretable risk stratification after PVC ablation. These models are suitable for integration into electronic health record dashboards, with external validation and local threshold optimization as next steps.

## 1. Introduction

Machine learning has transformed cardiovascular prognostication; however, existing models largely target peri-procedural complications [1], recurrence [2], or immediate procedural success after catheter ablation [3]. Premature ventricular contractions (PVCs), though often considered benign, harbor latent long-term risks: patients who undergo ablation remain susceptible to cerebrovascular events and cumulative excess mortality, hazards seldom quantified. Current literature provides limited insight into heart failure and death trajectories after PVC ablation and rarely interrogates the clinical determinants that drive these outcomes, limiting clinicians’ ability to deliver truly personalised follow-up care.

To address this gap, we analyze a nationwide cohort of PVC ablation patients to identify key predictors of three-year heart failure and mortality and to benchmark state-of-the-art machine-learning algorithms against conventional statistical methods. By elucidating how patient variables interact to shape long-term prognosis, we aim to deliver a practical risk-stratification tool that can be embedded within electronic health record dashboards, empowering clinicians to implement proactive interventions and optimise resource allocation while advancing precision cardiology.

### 1.1. Predicting Mortality

Across cardiovascular and inpatient settings, machine learning models have repeatedly improved mortality prediction compared with conventional approaches. In cardiac surgery, logistic regression and XGBoost each showed strong postoperative discrimination in CABG cohorts [4]. Using dynamic or admission EHR data, models anticipated mortality risk ahead of time in acute cardiovascular care and general medical wards [5,6,7]. Population- and disease-specific studies likewise reported gains with tree-based methods and deep learning, including random forest and deep models that outperformed traditional baselines, and strong signal in myocardial infarction and spontaneous coronary artery dissection cohorts [8,9,10,11]. Emergency department applications further demonstrated early risk stratification for high-acuity patients [12]. Together, these results justify our comparator set that spans interpretability and performance, with logistic regression as a transparent benchmark and tree-based ensembles and gradient boosting as state-of-the-art candidates. Notably, prior work has seldom targeted long-term outcomes after PVC ablation, which defines the clinical gap addressed here.

### 1.2. Predicting Heart Failure

Across heart failure (HF) prediction tasks, machine learning consistently outperforms traditional approaches using tabular clinical data. Tree-based ensembles, particularly Random Forest, have shown robust discrimination across heterogeneous datasets and feature sets [13,14]. Comparative studies further indicate advantages of ML over standard cardiovascular risk scores or conventional baselines [15,16]. Gradient boosting methods extend these gains, with XGBoost achieving strong performance in early detection from ECG-derived features [17]. To address the class imbalance that is typical in HF outcomes, studies applying SMOTE reported improved sensitivity without unacceptable loss of specificity in chronic HF cohorts [18]. Hybrid and voting ensembles can yield additional accuracy but may compromise interpretability and stability across settings [19,20,21]. Taken together, prior evidence supports a comparator portfolio centered on interpretable logistic regression and strong tree-based learners, including Random Forest and gradient boosting. What remains underexplored is long-term HF risk after premature ventricular contraction ablation, which defines the clinical gap addressed by our study.

### 1.3. Contributions and Challenges of Machine Learning in Clinical Medicine

Despite encouraging gains in risk prediction, the impact of machine learning on bedside decision-making remains limited. Opacity of many models reduces clinician trust and hinders adoption, frequently characterized as black-box behavior [22,23,24]. Transportability and stability are challenged by data heterogeneity, label noise, multimodal integration, and tuning sensitivity, all of which can induce overfitting [25,26,27,28,29]. Privacy and governance further constrain data sharing, and even federated approaches show inconsistent cross-institutional performance [30]. Collectively, these issues limit real-world utility despite strong retrospective metrics [31]. To address interpretability in the present study, we predefine model-agnostic explanations using Shapley additive explanations (SHAP) to quantify feature contributions and directionality at both cohort and patient levels, reported alongside discrimination metrics. This design aims to retain predictive performance while producing transparent, auditable outputs that support post-ablation risk communication and clinical decision support.

### 1.4. Features

Prior HF is the strongest predictor of subsequent HF hospitalization and readmission, with published cohorts reporting an approximately 30-percent readmission rate at one year and about 50 percent at three years [32]. Age is consistently associated with higher HF risk and readmission in population and clinical studies, including evidence identifying age as a leading determinant of rehospitalization [33]. Male sex, hypertension, diabetes mellitus, and chronic kidney disease are repeatedly linked to adverse HF outcomes across observational cohorts and registries [34,35,36]. Chronic kidney disease(CKD) relates to HF progression through fluid overload, ventricular hypertrophy, and uremic myocardial injury, and is associated with higher rehospitalization rates [37,38]. Based on this body of evidence, these covariates were predefined as core predictors for model development and evaluation in the present study.

### 1.5. Research Gap and Contribution

Although machine learning has improved prognostic modeling in cardiology, its application to patients undergoing PVC ablation remains underexplored. Prior work rarely addresses long-term outcomes after ablation, and few studies provide interpretable models tailored to this population. As a result, clinicians lack evidence-based tools for individualized follow-up focused on three-year heart failure and all-cause mortality.

We analyzed a nationwide cohort of PVC ablation patients and developed models to predict three-year heart failure and mortality. Class imbalance was mitigated using SMOTE and ROSE within cross-validation. We benchmarked logistic regression against tree-based learners, including CART, Random Forest, XGBoost, and LightGBM, and reported both ROC-AUC and PR-AUC based on out-of-fold predictions. To support clinical uptake, we quantified feature contributions and directionality using SHAP values.

This work contributes by quantifying long-term risk after PVC ablation, comparing modern machine learning with a transparent logistic baseline under rigorous evaluation, and providing model explanations that enable practical risk stratification in routine care.

## 2. Materials and Methods

The National Health Insurance Research Database (NHIRD), managed by the Health and Welfare Data Science Center under the Ministry of Health and Welfare in Taiwan, encompasses comprehensive claims data and detailed information on health services covered by the National Health Insurance (NHI) program. Launched in 1985, the NHI program now covers over 99% of Taiwan’s population. Specifically, the NHIRD collects medical records from 27 medical centers, including nine public and eighteen private institutions, and also covers a nationwide network of regional hospitals, district hospitals, and primary care clinics, thereby providing comprehensive coverage of healthcare services for Taiwan’s 23 million residents. The NHIRD collects a wide array of data, including demographic information, outpatient care, outpatient visit records, hospital admissions, dental services, surgeries, prescriptions, disease status, and dialysis histories.

In this study, we utilized NHIRD data to identify patients aged over 18 years who diagnosed with premature ventricular contractions (PVCs) and subsequently underwent catheter ablation. The index date was defined as the date of the ablation procedure. Disease definitions were classified according to the International Classification of Diseases, 9th and 10th Revisions, Clinical Modification (ICD-9-CM, ICD-10-CM), and procedural codes were identified through the procedure codes defined by the Ministry of Health and Welfare in Taiwan. The collected data included demographic and clinical information such as age, gender, comorbidities, and medications.

### 2.1. Collection of Demographics and Medical History

Baseline demographic and clinical data for enrolled patients were extracted from medical records and from medical records available at the time of study enrollment. Demographic data included age, gender, and comorbidities, such as ventricular tachycardia (VT), Acute Coronary Syndrome (ACS), hypertension (HTN), diabetes mellitus (DM), dyslipidemia, heart failure (HF), coronary artery disease (CAD), acute myocardial infarction (AMI), cerebrovascular disease (CVA), chronic kidney disease (CKD), chronic obstructive pulmonary disease (COPD) or asthma, end-stage renal disease (ESRD), malignancy, rheumatic disease, and moderate or severe liver disease (LD). Medication records encompassed various antiarrhythmic drugs classified as Class I (Ia, Ib, Ic), Class II (beta-blockers), Class III, and Class IV (calcium channel blockers).

### 2.2. Study Design and Participants

**Figure 1:**
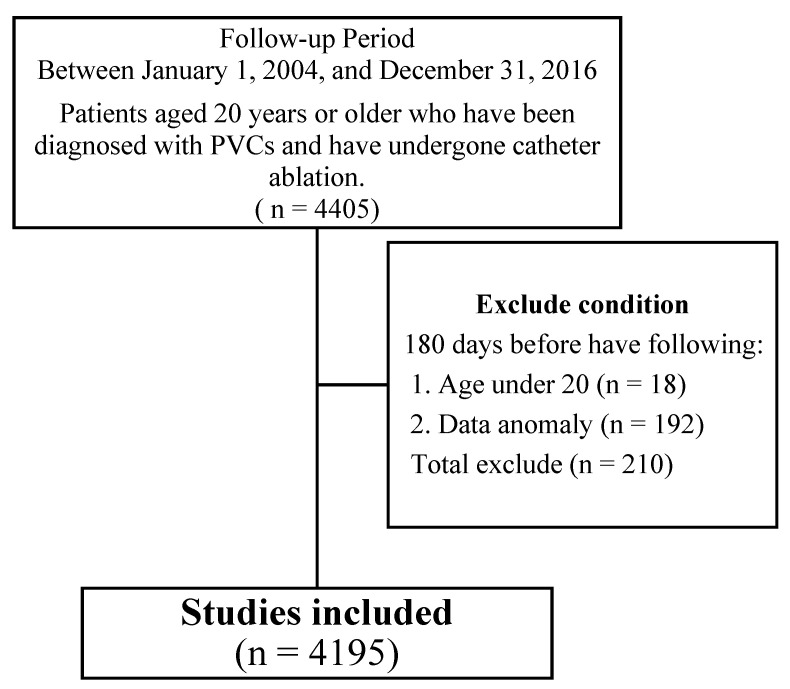
PRISMA flow diagram.

The cohort assembly and exclusions are summarized in a PRISMA style flow diagram (Figure 1), which outlines identification of eligible adults with PVC ablation, application of the prespecified 180 day exclusion window, and derivation of the final analytic cohort.

We conducted a nationwide retrospective cohort study, including adult patients who were diagnosed with PVCs and underwent catheter ablation between 1 January 2004, and 31 December 2016. Health records of the enrolled patients were extracted from the NHIRD. Inclusion criteria required patients to have a diagnosis of PVC prior to undergoing catheter ablation. Exclusion criteria included patients with any of the following conditions within 180 days before enrollment: atrial fibrillation, atrial flutter, paroxysmal supraventricular tachycardia (PSVT), and other cardiac arrhythmias. Enrolled patients were followed until occurrence of study outcomes, loss to follow-up, or 31 December 2021.

### 2.3. Baseline Statistic Analysis

Table 1 presents baseline characteristics of 4195 patients with PVC who underwent transcatheter radiofrequency ablation for arrhythmia. Of these patients, 50.6% were female, with a mean age of 52.38 years (SD = 14.67).

Comorbidities were common. HF occurred in 7.3%, VT in 5.6%, ACS in 2.5%, and CAD in 9.1% of patients, and 1.3% had PVD. HTN was the most common comorbidity, affecting 33.8% of the patients. DM was reported in 11.5% of the cases, and hyperlipidemia was noted in 23.5% of the patients.

Other notable comorbidities included COPD/Asthma in 10.1% of patients, CKD in 4.6%, and ESRD in 3.4%. Malignancy was present in 4.4% of the cohort, while 6.0% had a history of CVA. Rheumatic disease was noted in 1.6% of the patients, and LD (7.2%).

Medication use was common with 53.1% of patients taking beta-blockers. Class I antiarrhythmic drugs (AAD) Ia and Ib were used by 17.3%, and Class I AAD Ic by 23.5% of the patients. Class III AADs were used by 8.0% of the cohort, and calcium channel blockers (CCB) were prescribed to 45.8% of the patients.

The baseline characteristics indicate a diverse patient population with significant comorbidities and a broad range of medication usage. This diverse profile provides a robust foundation for subsequent machine learning analyses aimed at predicting post-ablation outcomes.

### 2.4. Method

This study used SAS Enterprise Guide 8.3 and R 4.3.1 for all analyses. SAS was employed for data extraction tasks, while R was used for data analysis and machine learning applications. In this study, we encountered significant data imbalance issues in both outcome variables (heart failure within three years post-surgery and mortality within three years) and aimed to address them by applying machine learning techniques to predict the outcomes of these variables.

**Figure 2:**
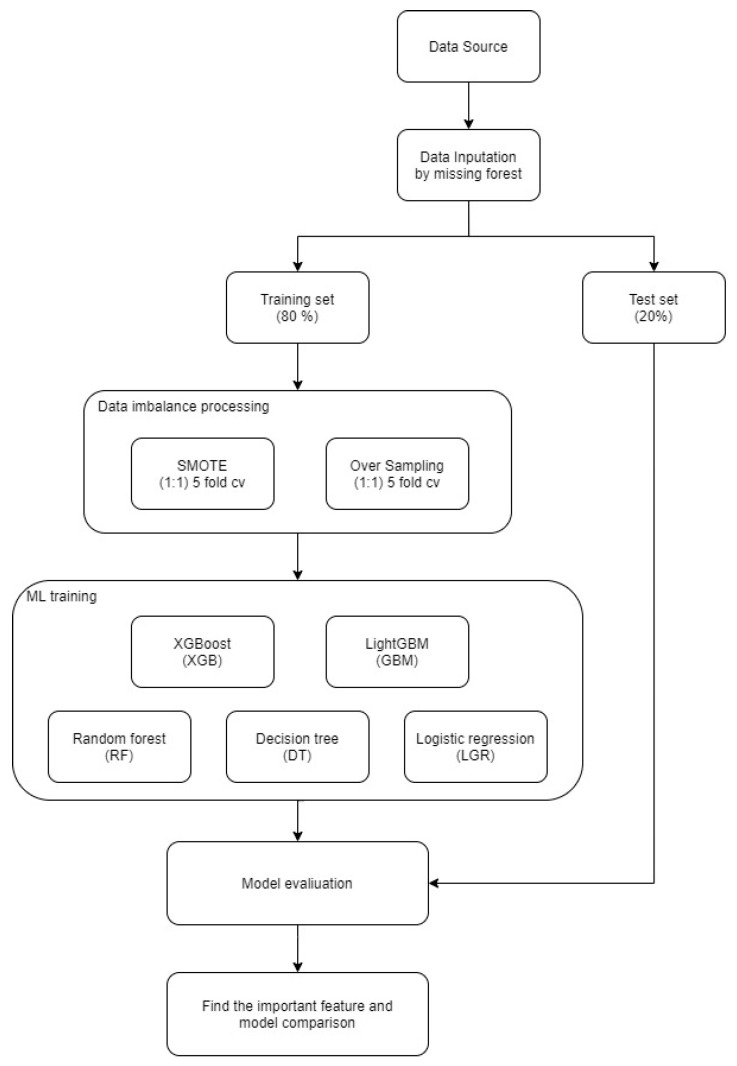
Design of Data Analysis Processes.

The end-to-end workflow, including missForest imputation, stratified train-test splitting, class rebalancing, model training, and performance evaluation, is summarized in Figure 2.

Recognizing that data imbalance may adversely affect the predictive performance of the models, we employed SMOTE and Random Over-Sampling methods (ROSE) separately to adjust the ratio of the dependent variable to balance positive and negative samples at a 1:1 ratio during model training. Class imbalance was handled with two oversampling approaches that create synthetic observations using different mechanisms. SMOTE generates new minority samples by linear interpolation between a target minority case and its k nearest minority neighbors. This increases minority density while preserving local feature geometry [39]. ROSE uses a smoothed bootstrap that samples from class-conditional kernel distributions centered on observed points, especially near the decision boundary, injecting small noise to reduce overfitting and overlap bias [40,41]. Both methods were applied only to the training folds, after which models were fitted and evaluated on untouched validation folds. We implemented the SMOTE package by Branco et al. [42], and the ROSE method by Lunardon et al. [41].

We compared five model families to span the pragmatic accuracy–interpretability spectrum for tabular clinical data. Logistic regression served as the transparent benchmark that yielded calibrated probabilities and remains the reference in cardiovascular risk modeling [43]. The CART decision tree provides human-readable splits to capture simple nonlinearity and interactions, facilitating bedside communication; however, variance can be high [44,45]. Random Forest aggregates many decorrelated trees, reducing variance and generally performing well on structured EHR features [46]. XGBoost and LightGBM are gradient-boosting frameworks that iteratively add shallow trees with regularization and efficient split finding, often delivering state-of-the-art discrimination in cardiology datasets. Prior studies in cardiac surgery and heart failure have shown that tree-based ensembles outperform logistic regression for mortality prediction, and multiple cohorts have reported top performance for gradient-boosting models [47]. For example, ML models improved mortality risk prediction after cardiac surgery compared with logistic regression. In heart failure cohorts, XGBoost achieved the highest discrimination among common classifiers. LightGBM has been successfully used for 1- to 3-year mortality risk stratification in chronic heart failure comorbid with atrial fibrillation [4,48,49,50]. Decision-tree–based models have also been applied for coronary artery disease detection, supporting the inclusion of CART and its ensembles. This portfolio strikes a balance between clinical interpretability and modern boosting accuracy, enabling unified SHAP-based explanations across tree models [6,51].

A stratified fivefold cross-validation scheme was employed to mitigate overfitting and ensure generalizable evaluation. The dataset was randomly partitioned into five equal subsets; each subset served once as the holdout test set while the remaining four subsets formed the training corpus, producing five independent training evaluation cycles. Averaging performance indices across folds reduced variance and yielded a stable estimate of predictive capacity while maintaining reasonable computational load. Hyperparameter optimization of the ensemble learners XGBoost and LightGBM was executed with GridSearchCV nested within the same cross-validation loop, guaranteeing that tuning bias did not contaminate the test folds. Appendix A and Table A1 and Table A2 provide a detailed summary of the selected parameters.

We evaluated model performance under class imbalance using a unified protocol. For each algorithm and resampling strategy, we performed 5-fold cross-validation, obtaining held-out predictions per fold to avoid optimism bias. From these out-of-fold scores, we computed Accuracy, Sensitivity, Specificity, F1, and ROC-AUC, reporting the mean and standard deviation across folds to summarize central tendency and variability. Discrimination in the imbalanced setting was further characterized by precision–recall analysis. Within each fold we swept the decision threshold from 0 to 1 to trace the precision–recall (PR) curve, then pooled out-of-fold predictions to generate one PR curve per model. Area under the PR curve (AUPRC) was obtained from the empirical PR curve via stepwise trapezoidal integration, with the dashed horizontal line denoting outcome prevalence as a no-skill baseline [52,53]. Formal pairwise comparisons of ROC-AUC used DeLong’s nonparametric test for correlated curves on the pooled out-of-fold predictions, with two-sided *p*-values and Holm adjustment for multiplicity [54]. All analyses were conducted in R using the pROC package, with statistical significance set at α = 0.05 [55].

## 3. Results

### 3.1. Heart Failure in 3 Years

In the three-year heart failure prediction analysis, the combination of ROSE and the LightGBM model demonstrated superior overall performance. As shown in Table 2, this approach achieved an AUC of 0.822, a sensitivity of 0.735, and a specificity of 0.784, indicating strong discriminative power and a balanced trade-off between false positives and false negatives. The ROSE-based logistic regression model also performed competitively, with an AUC of 0.817 and a specificity of 0.813, showing particular strength in identifying non–heart failure cases. In contrast, the SMOTE-based decision tree model achieved the highest specificity (0.900) but a relatively low sensitivity (0.440), suggesting potential under-detection of heart failure events.

Building on the point estimates in Table 2, which suggest that LightGBM–ROSE and logistic–ROSE offer the best balance of AUC, sensitivity, and specificity while CART–SMOTE trades sensitivity for specificity, we formally tested whether these apparent gaps were statistically meaningful. Pairwise DeLong tests on pooled out-of-fold predictions (Table A3) showed no significant AUC differences among LightGBM–ROSE, logistic–ROSE, and LightGBM–SMOTE (*p*-values 0.796, 0.615, and 0.415 in the relevant contrasts), indicating a top-performing cluster rather than a single dominant model; each of these models, however, significantly exceeded CART- and XGB-based variants in AUC, for example, LightGBM–ROSE vs. Cart–ROSE *p* = 0.001 and versus XGB–ROSE *p* = 0.039, while differences versus RF–based models were small or borderline.

**Figure A1:**
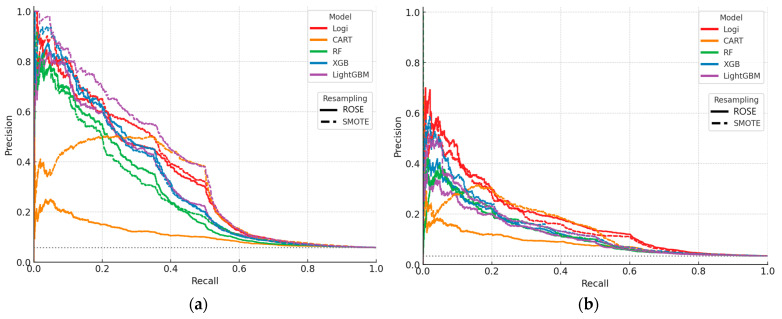
(**a**) PR curves for three-year heart failure. (**b**) PR curves for three-year mortality. Curves are colored by model and line style indicates oversampling method (ROSE vs. SMOTE). A dashed horizontal line marks the positive class prevalence.

Recognizing that ROC-AUC can understate performance in the presence of class imbalance, we then examined precision–recall behavior. Figure A1a shows that all models performed above the prevalence baseline, and LightGBM–ROSE achieves the highest AUPRC with a smoother precision decline and clear dominance in the clinically relevant recall band of 0.2 to 0.5. Taken together, the DeLong test results confirms that LightGBM–ROSE is statistically tied with the leading group in terms of ROC-AUC, and the AUPRC advantage provides incremental clinically actionable support for selecting LightGBM–ROSE as the primary model for risk stratification, with logistic-ROSE and LightGBM–SMOTE as closely competitive alternatives.

**Figure 3:**
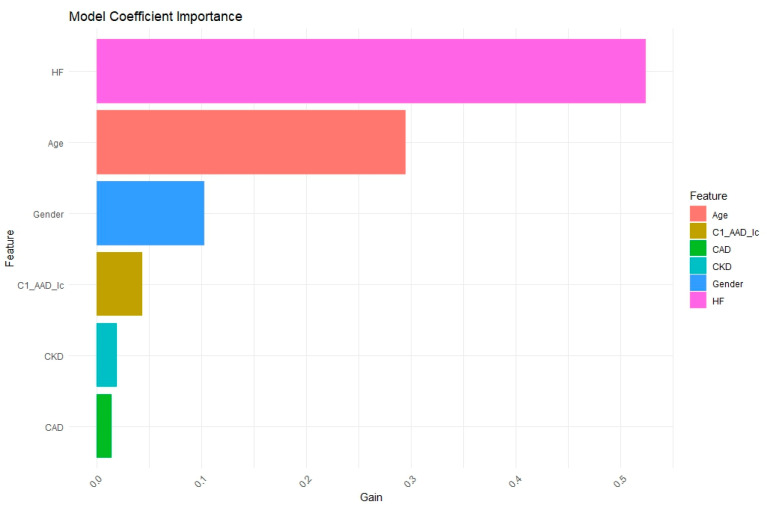
Key factors for predicting three-year heart failure using oversampling LightGBM. Feature importance was computed as LightGBM gain, averaged across 5-fold cross-validation and normalized to a 0 to 1 scale. Note: HF = history of heart failure; CKD = chronic kidney disease; CAD = coronary artery disease; C1_AAD_Ic = class Ic antiarrhythmic exposure.

**Figure A2:**
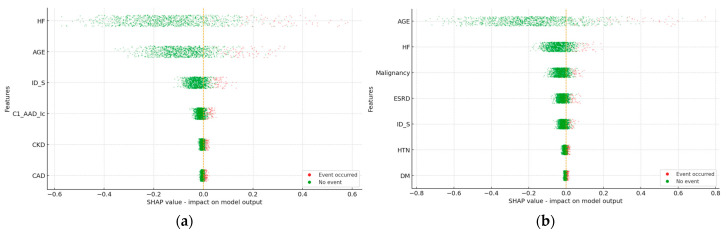
SHAP value analysis using the ROSE-augmented LightGBM model for three-year post-ablation outcomes. (**a**) Model interpretability analysis for predicting three-year heart failure risk. (**b**) Model interpretability analysis for predicting three-year mortality risk.

As illustrated in Figure 3, feature importance analysis from the LightGBM model combined with ROSE revealed that a prior history of heart failure contributed most strongly to predicting three-year heart failure, followed by age. Sex had a moderate influence, while chronic kidney disease (CKD), coronary artery disease (CAD), and exposure to class Ic antiarrhythmic drugs (C1_AAD_Ic) provided secondary but meaningful contributions, highlighting key aspects of model interpretability. Finally, SHAP analysis in Figure A2a confirmed that prior heart failure, older age, CKD, and CAD were the principal factors associated with increased postoperative risk of heart failure within three years.

### 3.2. Mortality in 3 Years

In the three-year mortality risk prediction analysis, all machine learning models demonstrated stable and clinically meaningful predictive performance. As shown in Table 3, the decision tree model combined with the SMOTE oversampling method achieved the highest overall accuracy (0.908) and specificity (0.927), indicating excellent capability for identifying non-death cases. However, its sensitivity was relatively low (0.403), suggesting a potential risk of missed death predictions. In contrast, the logistic regression model combined with ROSE achieved a sensitivity of 0.847 and an AUC of 0.886, exhibiting strong discriminative power and reliable overall performance in predicting mortality risk. The LightGBM model combined with ROSE also performed remarkably well, attaining an AUC of 0.882 and a sensitivity of 0.879, which demonstrates its effectiveness in identifying individuals at high risk of death. Although its specificity (0.793) was slightly lower, it maintained a superior balance across key evaluation metrics. Considering accuracy, sensitivity, specificity, and AUC collectively, both the Logistic Regression–ROSE and LightGBM–ROSE models exhibited the most balanced and robust performance, suggesting strong clinical feasibility and reproducibility for three-year mortality risk prediction. These models warrant further validation in broader populations to confirm their applicability and scalability.

To assess whether the apparent gaps in Table 3 were statistically significant, we compared ROC-AUCs using pairwise DeLong tests on the pooled out-of-fold predictions (Table A4). LightGBM–ROSE, logistic–ROSE, LightGBM–SMOTE, and logistic–SMOTE were not statistically different from one another (*p*-values 0.796, 0.415, 0.615, and 0.591 across the relevant contrasts), indicating a top-performing cluster rather than a single dominant model. Each of these models significantly exceeded CART- and XGB-based variants in AUC, for example, LightGBM–ROSE vs. Cart–ROSE *p* = 0.001 and vs. XGB–ROSE *p* = 0.039, while differences versus RF-based models were small or borderline at most (for example LightGBM–ROSE vs. RF–SMOTE *p* = 0.053 and logistic–ROSE vs. RF–SMOTE *p* = 0.045). Therefore, AUC alone does not isolate a unique winner, which motivates examining precision–recall behavior in the next paragraph to adjudicate performance under class imbalance.

As illustrated in Figure A1b, all models achieved PR curves above the positive class prevalence line, confirming substantial discriminative capability. Among them, the LightGBM model combined with ROSE yielded the highest overall AUPRC and maintained relatively higher and more stable precision within the mid-to-high recall range, which is particularly advantageous for detecting rare mortality events. Its curve showed dominance across most recall intervals, indicating a more favorable trade-off between precision and recall and greater clinical applicability, thereby positioning it as the most representative predictive framework in this study.

**Figure 4:**
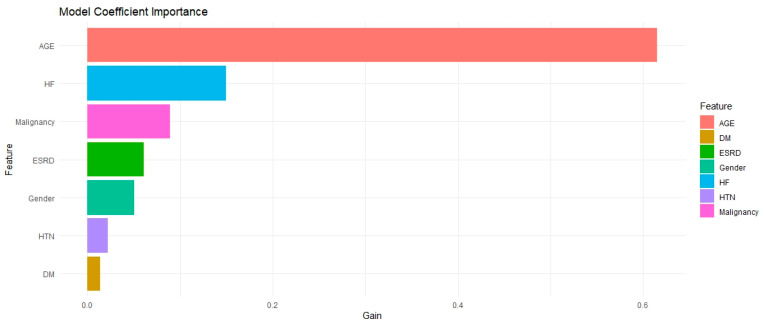
Key Factors of the Model Predicting Future Three-Year Mortality Using Random Over-sampling LightGBM for Data Modeling.

Furthermore, as shown in Figure 4, the feature importance analysis based on ROSE-LightGBM model revealed that age was the most influential predictor of mortality risk, with an importance score significantly higher than those of other variables, emphasizing the critical role of aging as a determinant of long-term mortality. Following age, preexisting HF contributed substantially to mortality prediction, reflecting the prognostic impact of cardiac dysfunction. Malignancy also exhibited a high feature importance, indicating the detrimental effect of cancer on overall survival, while ESRD and sex were moderately influential yet contributed meaningfully to prediction outcomes. Consistently, the SHAP value analysis in Figure A2b confirmed that age, prior HF, malignancy, and ESRD exerted the greatest positive effects on mortality prediction. Overall, the ROSE–LightGBM model not only demonstrated superior predictive accuracy and stability but also aligned with clinical evidence in feature interpretability, underscoring its potential clinical value as a robust predictive tool for mortality risk stratification.

## 4. Discussion

The application of ML models in healthcare, particularly for predicting heart failure and mortality, is increasingly gaining traction due to their ability to handle large and complex datasets more effectively than traditional statistical methods. This study’s findings align with existing literature that highlights the superiority of ML techniques in predictive accuracy.

### 4.1. Data Imbalance and Discrimination Beyond ROC

Class imbalance posed a key analytic risk given the rarity of outcomes such as heart failure and mortality. We mitigated this by balancing the training data with SMOTE and ROSE, which reduced majority-class bias and improved model performance. Because ROC AUC can be optimistic for minority classes, we also reported precision–recall curves and AUPRC. All models performed above the prevalence baseline, with LightGBM trained on ROSE achieving the highest AUPRC and a smoother precision decay across mid-range recall, indicating stronger case finding under scarcity and clear operational value for post-ablation surveillance.

### 4.2. Model Selection and Evaluation

Across five algorithms, LightGBM and logistic regression combined with data balancing (SMOTE or ROSE) delivered the strongest discrimination by ROC AUC. Pairwise DeLong tests on pooled out-of-fold predictions showed no significant differences among LightGBM–ROSE, Logistic–ROSE, LightGBM–SMOTE, and Logistic–SMOTE, indicating a top cluster rather than a single winner. CART and several XGBoost variants were significantly lower, and random forest was generally intermediate. Considering precision–recall results, LightGBM–ROSE is preferable for sensitivity-oriented use cases, while Logistic–ROSE remains a transparent baseline with competitive discrimination and calibrated probabilities familiar to clinicians. These findings support future integration into clinical decision support for real-time risk assessment.

### 4.3. Clinical Implications and Deployment

This study identified age, history of heart failure, and malignancy as key predictors, suggesting enhanced surveillance for patients undergoing VPC ablation. Leveraging the high-dimensional data in electronic medical records, the best-performing model employed recall-biased thresholds during outpatient and postoperative follow-up to reduce missed positives and direct flagged cases toward targeted assessment and treatment optimization. Risk quantiles supported capacity and follow-up planning, while SHAP provided auditable interpretation at both the population and individual levels, facilitating bedside discussions and shared decision-making. Because the predictive variables used are routinely available, the model can be seamlessly integrated into EHR dashboards, promoting earlier intervention and precise follow-up, with the potential to reduce the three-year risk of heart failure and mortality.

### 4.4. Limitations, Future Directions

This study has several qualifications. NHIRD is claims-based, which introduces coding misclassification and limits access to echocardiography, biomarkers, and electrophysiology parameters, so residual confounding is possible. Our cohort was drawn from Taiwan, therefore generalizability to other health systems requires external, preferably multi center validation. Event prevalence can shift over time and across sites, which affects precision–recall behavior and alert thresholds, so periodic recalibration and threshold review are advisable. In this round we added precision–recall analyses as requested; formal calibration plots and decision curve analysis were not included and remain appropriate targets for follow on work. Models were evaluated with cross-validated out-of-fold predictions; prospective testing is needed before deployment at scale.

Future work should validate these models across larger and more diverse populations and health systems, enrich predictors with more granular variables such as genomics and lifestyle factors, and deliver user friendly, clinician facing interfaces embedded in EHR dashboards. A pragmatic deployment plan should include performance monitoring, drift detection, scheduled recalibration, fairness and calibration audits, and decision analytic evaluation of net benefit.

In conclusion, carefully balanced data pipelines paired with advanced machine learning materially improved prognostic accuracy for patients undergoing VPC ablation. With external validation, calibration and decision curve analyses, prospective evaluation, and thoughtful workflow integration, these tools can support personalized and proactive care while informing resource allocation.

## 5. Conclusions

In a nationwide cohort of patients undergoing catheter ablation for premature ventricular contractions, addressing class imbalance with SMOTE and ROSE enabled robust three-year risk prediction for heart failure and all-cause mortality using routinely available tabular clinical data. Across five algorithms including Logistic Regression, Decision Trees, Random Forests, XGBoost, and LightGBM, LightGBM with ROSE achieved the highest AUC for heart failure at 0.822. For mortality, Logistic with ROSE and LightGBM with ROSE performed comparably well with AUCs of 0.886 and 0.882. SHAP analyses confirmed the central roles of age, prior heart failure, and malignancy with clinically consistent directionality, highlighting actionable factors for surveillance.

Pairwise DeLong tests on pooled out-of-fold predictions indicated that LightGBM ROSE and Logistic ROSE variants formed a high-performing cluster rather than a single definitive winner. Precision–recall analyses and AUPRC favored LightGBM ROSE for recall-oriented use cases, while Logistic ROSE remains an interpretable baseline familiar to clinicians. Embedding these models within electronic health record dashboards, coupled with site-specific threshold optimization and periodic performance review, offers a practical path to individualized follow up and timely interventions.

Importantly, combining logistic regression with modern ML techniques not only validates traditional linear risk factors but also uncovers non-linear patterns that conventional models may miss. This dual approach enhances the ability to detect subtle yet clinically meaningful predictors, enabling earlier recognition of high-risk patients. In clinical practice, such predictive models could be embedded within electronic health record dashboards to provide individualized risk assessments, support high-intensity monitoring or timely therapeutic interventions, and facilitate shared decision-making. Ultimately, these models can serve as practical decision-support tools, helping clinicians make more informed choices and potentially reducing the long-term burden of heart failure and mortality.

## Figures and Tables

**Table 1:** Baseline characteristics of PVC patient with Transcatheter Radiofrequency Ablation for Arrhythmia Surgery.

Features	Total Number (n = 4195)
Gender (Female)	2124 (50.6%)
Age	52.38 ± 14.67
Comorbidities (%)	
HF	308 (7.3%)
VT	234 (5.6%)
ACS	103 (2.5%)
CAD	382 (9.1%)
PVD	53 (1.3%)
HTN	1418 (33.8%)
DM	484 (11.5%)
Hyperlipidemia	986 (23.5%)
COPD	422 (10.1%)
CKD	195 (4.6%)
ESRD	141 (3.4%)
Malignancy	185 (4.4%)
CVA	251 (6.0%)
Rheumatic	67 (1.6%)
LD	303 (7.2%)
Medications (%)	
B_blocker	2226 (53.1%)
C1_AAD_Ia & Ib	724 (17.3%)
C1_AAD_Ic	987 (23.5%)
C3_AAD	335 (8.0%)
CCB	1922 (45.8%)

Note: HF = Heart failure; VT = ventricular tachycardia; ACS = Acute Coronary Syndrome; CAD = Coronary artery disease; PVD = Peripheral vascular disease; HTN = hypertension; DM = Diabetes mellitus; COPD = Chronic Obstruction Pulmonary Disease; CKD = Chronic kidney disease; ESRD = end stage renal disease; CVA = cerebrovascular accident; LD = moderate or severe liver disease; B_blocker = beta-blockers; C1_AAD_Ia & Ib = Class I antiarrhythmic drugs Ia, Ib; C1_AAD_Ic = Class I antiarrhythmic drugs Ic; C3_AAD = Class III antiarrhythmic drugs; CCB = calcium channel blockers.

**Table 2:** Evaluation Metrics for Predicting Heart Failure Events within 3 Years After PVC Ablation Surgery Using Machine Learning.

Model & Data Processing Method	Accuracy	Sensitivity	Specificity	F1	AUC
Logi_ROSE	0.809 (0.085)	0.707 (0.081)	0.813 (0.093)	0.316 (0.041)	0.817 (0.012)
Cart_ROSE	0.816 (0.013)	0.493 (0.060)	0.836 (0.018)	0.236 (0.053)	0.665 (0.026)
RF_ROSE	0.756 (0.072)	0.663 (0.077)	0.760 (0.080)	0.241 (0.027)	0.752 (0.021)
XGB_ROSE	0.753 (0.086)	0.504 (0.043)	0.768 (0.091)	0.197 (0.043)	0.584 (0.026)
LightGBM_ROSE	0.782 (0.051)	0.735 (0.043)	0.784 (0.055)	0.281 (0.036)	0.822 (0.018)
Logi_SMOTE	0.824 (0.066)	0.688 (0.072)	0.831 (0.072)	0.321 (0.032)	0.805 (0.026)
Cart_SMOTE	0.873 (0.005)	0.44 (0.069)	0.9 (0.011)	0.281 (0.037)	0.67 (0.03)
RF_SMOTE	0.724 (0.118)	0.707 (0.129)	0.723 (0.132)	0.243 (0.047)	0.758 (0.025)
XGB_SMOTE	0.757 (0.065)	0.61 (0.048)	0.765 (0.069)	0.227 (0.03)	0.675 (0.043)
LightGBM_SMOTE	0.748 (0.093)	0.726 (0.077)	0.748 (0.104)	0.257 (0.038)	0.801 (0.018)

Note: F1 = F1 score; AUC = area under curve; Logi = Logistic regression; Cart = Decision tree; RF = random forest; ROSE = Random over-sampling; SMOTE = Synthetic Minority Oversampling Technique, XGB = Xgboost.

**Table 3:** Evaluation Metrics for Predicting Mortality Events within 3 Years After PVC Ablation Surgery Using Machine Learning.

Model & Data Processing Method	Accuracy	Sensitivity	Specificity	F1	AUC
Logi_ROSE	0.826 (0.046)	0.847 (0.084)	0.826 (0.048)	0.253 (0.076)	0.886 (0.047)
Cart_ROSE	0.897 (0.015)	0.483 (0.134)	0.912 (0.008)	0.231 (0.036)	0.698 (0.071)
RF_ROSE	0.807 (0.069)	0.773 (0.081)	0.807 (0.072)	0.219 (0.048)	0.831 (0.047)
XGB_ROSE	0.735 (0.303)	0.61 (0.171)	0.74 (0.311)	0.205 (0.114)	0.68 (0.18)
LightGBM_ROSE	0.797 (0.032)	0.879 (0.06)	0.793 (0.033)	0.224 (0.059)	0.882 (0.044)
Logi_SMOTE	0.78 (0.084)	0.877 (0.095)	0.776 (0.091)	0.221 (0.064)	0.878 (0.046)
Cart_SMOTE	0.908 (0.018)	0.403 (0.098)	0.927 (0.022)	0.227 (0.075)	0.665 (0.048)
RF_SMOTE	0.769 (0.071)	0.847 (0.038)	0.767 (0.074)	0.204 (0.069)	0.845 (0.042)
XGB_SMOTE	0.826 (0.062)	0.625 (0.11)	0.832 (0.065)	0.2 (0.062)	0.669 (0.067)
LightGBM_SMOTE	0.764 (0.086)	0.889 (0.057)	0.759 (0.091)	0.208 (0.047)	0.87 (0.038)

Note: F1 = F1 score; AUC = area under curve; Logi = Logistic regression; Cart = Decision tree; RF = random forest; ROSE = Random over-sampling; SMOTE = Synthetic Minority Oversampling Technique, XGB = Xgboost.

## Data Availability

The data utilized in this study were obtained from Taiwan’s National Health Insurance Research Database (NHIRD), a restricted-access resource. Access to the NHIRD requires formal application and approval from the Health and Welfare Data Science Center, Ministry of Health and Welfare, Taiwan (https://dep.mohw.gov.tw/DOS/np-2497-113.html, accessed on 13 October 2025). To protect patient privacy, all personal identifiers within the database are encrypted and de-identified.

## Abbreviations

The following abbreviations are used in this manuscript:

PVC	Premature Ventricular Contraction
ML	Machine learning
HF	Heart failure
VT	ventricular tachycardia
ACS	Acute Coronary Syndrome
CAD	Coronary artery disease
PVD	Peripheral vascular disease
HTN	hypertension
DM	Diabetes mellitus
COPD	Chronic Obstruction Pulmonary Disease
CKD	Chronic kidney disease
ESRD	end stage renal disease
CVA	cerebrovascular accident
LD	moderate or severe liver disease
B_blocker	beta-blockers
C1_AAD_Ia & Ib	Class I antiarrhythmic drugs Ia, Ib
C1_AAD_Ic	Class I antiarrhythmic drugs Ic
C3_AAD	Class III antiarrhythmic drugs
CCB	Calcium channel blockers
F1	F1 score
AUC	Area under curve
Logi	Logistic regression
Cart	Decision tree
RF	Random forest
ROSE	Random over-sampling
SMOTE	Synthetic Minority Oversampling Technique
XGB	Xgboost
SHAP	Shapley additive explanations

## Appendix A

**Table A1:** XGBoost model Hyperparameters.

Hyperparameters	Setting
colsample_bytree	0.8
subsample	0.8
booster	gbtree
max_depth	10
eta	0.01
eval_metric	auc
eval_metric	error
objective	binary: logistic
gamma	0.01
lambda	2
min_child_weight	1

**Table A2:** LightGBM model Hyperparameters.

Feature	Racy
num_leaves	3
nthread	1
metric	auc
metric	binary_error
objective	binary
min_data	1
learning_rate	0.1

## Appendix B

**Table A3:** Predicting three-year heart failure model AUC Delong test *p*-value matrix. green indicates that the two methods show a statistically significant difference based on the test results, whereas orange indicates no significant difference.

	Logi _ROSE	Cart _ROSE	RF _ROSE	XGB _ROSE	LightGBM _ROSE	Logi_ SMOTE	Cart_ SMOTE	RF_ SMOTE	XGB_ SMOTE	LightGBM _SMOTE
Logi_ ROSE	1.000	0.001	0.021	0.036	0.796	0.615	0.000	0.045	0.000	0.316
Cart_ ROSE	0.001	1.000	0.003	0.781	0.001	0.001	0.193	0.002	0.256	0.001
RF_ ROSE	0.021	0.003	1.000	0.085	0.024	0.033	0.000	0.381	0.001	0.049
XGB_ ROSE	0.036	0.781	0.085	1.000	0.039	0.041	0.831	0.071	0.866	0.051
LightGBM _ROSE	0.796	0.001	0.024	0.039	1.000	0.793	0.000	0.053	0.000	0.415
Logi_ SMOTE	0.615	0.001	0.033	0.041	0.793	1.000	0.000	0.078	0.000	0.591
Cart_ SMOTE	0.000	0.193	0.000	0.831	0.000	0.000	1.000	0.000	0.850	0.000
RF_ SMOTE	0.045	0.002	0.381	0.071	0.053	0.078	0.000	1.000	0.001	0.122
XGB_ SMOTE	0.000	0.256	0.001	0.866	0.000	0.000	0.850	0.001	1.000	0.001
LightGBM _SMOTE	0.316	0.001	0.049	0.051	0.415	0.591	0.000	0.122	0.001	1.000

**Table A4:** Predicting three-year mortality model AUC Delong test *p*-value matrix. green indicates that the two methods show a statistically significant difference based on the test results, whereas orange indicates no significant difference.

	Logi _ROSE	Cart _ROSE	RF _ROSE	XGB _ROSE	LightGBM _ROSE	Logi_ SMOTE	Cart_ SMOTE	RF_ SMOTE	XGB_ SMOTE	LightGBM _SMOTE
Logi_ ROSE	1	0.001	0.021	0.036	0.796	0.615	0.000	0.045	0.000	0.316
Cart_ ROSE	0.001	1	0.003	0.781	0.001	0.001	0.193	0.002	0.256	0.001
RF_ ROSE	0.021	0.003	1	0.085	0.024	0.033	0.000	0.381	0.001	0.049
XGB_ ROSE	0.036	0.781	0.085	1	0.039	0.041	0.831	0.071	0.866	0.051
LightGBM _ROSE	0.796	0.001	0.024	0.039	1	0.793	0.000	0.053	0.000	0.415
Logi_ SMOTE	0.615	0.001	0.033	0.041	0.793	1	0.000	0.078	0.000	0.591
Cart_ SMOTE	0.000	0.193	0.000	0.831	0.000	0.000	1	0.000	0.850	0.000
RF_ SMOTE	0.045	0.002	0.381	0.071	0.053	0.078	0.000	1	0.001	0.122
XGB_ SMOTE	0.000	0.256	0.001	0.866	0.000	0.000	0.850	0.001	1	0.001
LightGBM _SMOTE	0.316	0.001	0.049	0.051	0.415	0.591	0.000	0.122	0.001	1
